# Simulated Assessment of the Impact of Climate Change on the Potential Distribution Range of Four *Taxus* Species in China

**DOI:** 10.3390/plants15050721

**Published:** 2026-02-27

**Authors:** Quanlong Jin, Yu Gao, Yuandong Hu

**Affiliations:** 1 College of Landscape Architecture, Northeast Forestry University, Harbin 150040, China; 2023110568@nefu.edu.cn; 2 Forest Protection Research Institute of Heilongjiang, Harbin 150030, China; gaoyu811127@163.com; 3 XAUAT-UWA International Joint Laboratory on Urban Biodiversity and Design, Xi’an University of Architecture and Technology, Xi’an 710055, China

**Keywords:** *Taxus*, climate change, MaxEnt model, potential geographic distribution pattern, suitable habitat

## Abstract

*Taxus*, a relic plant genus from the Tertiary period, contains taxane compounds that are crucial in anti-cancer drug development and have significant medicinal and ecological value. Evaluation of the potential distribution range and shifts for this genus considering global climate change is vital for conserving wild resources, supporting artificial propagation, and ensuring sustainable development. We analyzed the potential geographic distribution patterns and key environmental factors affecting four *Taxus* species (*Taxus cuspidata*, *Taxus wallichiana* var. *mairei*, *Taxus wallichiana*, and *Taxus wallichiana* var. *chinensis*) under current climate conditions and four shared socioeconomic pathways (SSP126, SSP245, SSP370, and SSP585) across three future periods (2050s, 2070s, and 2090s) using the regularization multiplier and feature combination parameters of the MaxEnt model. We also explored their responses to climate change over time. The area under the curve of models built using the ENMeval package exceeded 0.9, demonstrating high accuracy. Environmental analysis indicated that the coldest monthly minimum temperature was the main environmental factor influencing the species distribution, except in *Taxus cuspidata*, for which the human footprint was the primary factor. Currently, the habitats of the four *Taxus* species exhibit spatial variation, with *Taxus wallichiana* var. *chinensis* having the largest suitable area in China, covering approximately 200.89 × 10^4^ km^2^, accounting for 21.17% of China’s land area. Habitat trends varied under future climate scenarios, with the suitable area expanding for *Taxus wallichiana* and *Taxus wallichiana* var. *chinensis*, and showing expansion and contraction for *Taxus wallichiana* var. *mairei* and *Taxus cuspidata*. The distribution centroids were predicted to shift to higher latitudes over time, with *Taxus wallichiana* var. *chinensis* showing particularly clear migration trends. These results offer a vital reference for developing conservation strategies and introduction and cultivation initiatives for these *Taxus* species.

## 1. Introduction

According to the Sixth Assessment Report of the United Nations Intergovernmental Panel on Climate Change (IPCC), global climate change is accelerating at an unprecedented rate and scale, creating challenges for ecosystems and biodiversity [1,2]. Temperature increases, precipitation pattern changes, and more frequent extreme weather events cause ecological damage and species habitat loss and fragmentation [2]. As a result, the geographic ranges of many species have moved to higher latitudes or elevations, and some even face localized extinction risks [3]. Rare and endangered plants, which often have a narrow distribution and limited adaptability, are especially vulnerable to climate change. Their populations and habitats may continue to shrink, worsening the biodiversity crisis [1]. The IPCC warns that exceeding a 1.5 °C rise in global temperature will cause irreversible ecosystem damage and significantly increase extinction risks [4]. Therefore, studying the possible effects of climate change on species distribution patterns will clarify how ecosystems respond and provide a scientific basis for biodiversity conservation and adaptive management. This research has theoretical and practical implications for developing strategies to address global climate change.

Species distribution models (SDMs) are statistical or machine learning methods that predict the potential species distribution range by determining the relationship between environmental variables and the species distribution. Their core principle is based on niche theory, which suggests that the species distribution is limited by environmental conditions [5]. SDMs forecast the likelihood of species presence in unknown areas by building a correlation model using known species distribution points and environmental data [6]. These models are widely recognized as effective tools for predicting suitable habitats for species under climate change. Researchers have extensively utilized SDMs. For example, Thuiller et al. [7] used the BIOMOD model to project the distribution shifts in European alpine plant species due to climate change, providing scientific support for endangered plant conservation. Pearson et al. [8] employed the MaxEnt model to determine the impact of climate change on the distribution of European amphibians, revealing that the suitable habitats of some species would significantly decrease. Hijmans et al. [9] validated the use of SDMs in agricultural biodiversity conservation by comparing changes in the global suitability range of potatoes under different climate scenarios. Guisan et al. [10] systematically outlined the application framework of SDMs in ecology and conservation biology. These studies offer essential theoretical foundations and methodological guidance for using SDMs to assess the effects of climate change on the species distribution. Among various SDMs, the MaxEnt model is particularly popular due to its ability to maintain high predictive accuracy even with a limited sample size. Its advantage is its optimization of the weighting of environmental variables based on the principle of maximum entropy, which significantly reduces overfitting risks and effectively manages complex environmental interactions. It is widely used in ecology, conservation biology, and climate change research, providing a scientific basis for biodiversity conservation, ecosystem management, and developing climate change adaptation strategies.

The *Taxus* genus belonging to the Taxaceae family mainly consists of evergreen trees and shrubs, commonly called “yew” in Chinese folklore. Every part of the plant is valuable and has significant scientific research importance [11]. *Taxus* wood has uniform grain, a dense structure, high toughness, hardness, excellent elasticity, natural luster, and strong preservative qualities. As a high-quality industrial material, it is frequently used in detailed craftsmanship, such as carving, musical instruments, woodturning, and stationery [12]. The medicinal properties of *Taxus* were first recorded in the “*Compendium of Materia Medica*”, where it was noted to treat cholera and typhoid fever. “*Bencao Tui Chen*” also documented its medicinal uses, stating that “*Taxus* can be used medicinally to promote urination, regulate menstruation, and treat kidney diseases. The bark induces vomiting, while the wood and leaves do not” [13]. Pharmacological research and applications for *Taxus* species began when American chemists Wall and M.C. Wani first isolated taxol from *Taxus brevifolia* in 1971 and published its chemical structure [14]. In the 1980s, researchers in the US and Europe demonstrated the anti-cancer effects of taxol [15], establishing it as an effective treatment for breast and ovarian cancers. As a result, the medicinal value of *Taxus* species has received widespread attention, with global efforts focused on developing and applying this natural anti-cancer compound [16].

There are four species present in China: *Taxus cuspidata*, *Taxus wallichiana* var. *mairei*, *Taxus wallichiana*, and *Taxus wallichiana* var. *chinensis*. They are primarily distributed across Northeast, Southwest, Central, South, and East China [11]. As an important medicinal plant resource, *Taxus* species attract considerable attention from scientists and the medical community, but this presents challenges for conserving germplasm resources. Due to large-scale exploitation of *Taxus* species, most are now endangered. Wild *Taxus* populations face constraints to growth and reproduction, including imbalanced sex ratios, a scattered distribution, poor pollination environments resulting in low seed production, prolonged seed dormancy, low germination rates, slow seedling growth, low stress tolerance, weak population competitiveness, and strict habitat requirements that hinder natural regeneration [13]. External factors have accelerated the rapid decline of *Taxus* resources. These trees are shade-tolerant and have very specific habitat needs. Their seeds are often eaten by animals because of their sweet aril. Their overexploitation for timber and medicinal use has been ongoing, and human activities are altering forest ecosystems, degrading their growth environments [13].

In recent years, the use of species distribution models to assess the impact of climate change on the potential distribution of *Taxus* species has gained increasing attention and achieved notable progress. For example, Chen et al. [17] employed the MaxEnt model to predict the potential suitable habitat of *Taxus cuspidata* in China; Zhou et al. [18] employed the MaxEnt model to identify key environmental factors influencing the geographic distribution of *Taxus wallichiana* var. *chinensis* and predicted its highly suitable habitats in China; and Shu et al. [19] utilized the MaxEnt model to forecast the potential suitable distribution areas of *Taxus wallichiana* within the Tibet Autonomous Region, revealing its spatial distribution pattern and determining the key environmental factors affecting its distribution. However, existing studies predominantly focus on a single species, and as such lack systematic comparative and integrated analyses of how major Chinese yew species respond to climate change. Furthermore, many studies fail to fully optimize critical parameters of the MaxEnt model during modeling, such as regularization multiplier and feature combinations, which may compromise prediction accuracy. To better understand how current and future climate change might affect the distribution of *Taxus* species, this study focused on four key species in China. Using the MaxEnt SDM optimized by the ENMeval package, it simulated and predicted the possible geographic spread of these plants in China under current and future climate scenarios. The aim was to determine how climate change influences their range, forecast future distribution trends, and evaluate the potential impacts of climate change on suitable habitats. This study offers a scientific basis for developing both in situ and ex situ conservation strategies for *Taxus* species, supports the optimization of artificial introduction and cultivation zoning, and informs seed resource allocation plans. These efforts aim to prevent resource waste and economic losses due to indiscriminate introductions. Ultimately, this study seeks to promote the sustainable conservation and rational use of this rare and endangered plant resource.

## 2. Results

### 2.1. Optimal Parameters and Accuracy Validation for MaxEnt Models

The optimization results for MaxEnt model parameters (Table 1) showed that all corrected difference in Akaike Information Criterion (ΔAICc) values were zero, indicating that the optimized parameters effectively decreased both model complexity and goodness-of-fit. Using distribution point data and corresponding environmental variables for four *Taxus* species, optimal simulation parameters were set for each species to predict their current and future potential suitable habitats. The average AUC values for the prediction results were all above 0.9, and the average TSS values were all above 0.7, indicating that the model performed well in prediction.

### 2.2. Primary Environmental Factors Affecting the Distribution of Four Taxus Species

Using the jackknife method, we analyzed environmental factors influencing the potential geographic distributions of four *Taxus* species (Table 2). The environmental drivers affecting the distribution of different *Taxus* species varied. The environmental factors affecting the distribution of *Taxus cuspidata*, ranked by contribution rate, were the human footprint (Hfp), precipitation in the warmest quarter (Bio18), temperature seasonality (Bio4), topsoil basic saturation (T_BS), monthly mean diurnal temperature range (Bio2), elevation, and isothermality (Bio3), collectively accounting for 88.3% of the variation. Hfp exerted the strongest influence with a contribution rate of 39.1%. The environmental factors influencing the distribution of *Taxus wallichiana* var. *mairei*, ranked by contribution rate, were minimum temperature in the coldest month (Bio6), precipitation in the driest month (Bio14), mean temperature in the warmest season (Bio8), T_BS, monthly mean diurnal temperature range (Bio2), and Hfp. These six factors together explained 92.7% of the variation, with Bio6 having the greatest influence at 69%. The environmental factors affecting *Taxus wallichiana*, ranked by contribution rate, were Bio6, Bio8, and slope. Together, these three factors accounted for 85.1% of the variation, with Bio6 having the greatest impact at 66.7%. The environmental factors affecting the distribution of *Taxus wallichiana* var. *chinensis*, ranked by contribution rate, were Bio6, Hfp, and annual mean precipitation (Bio12). These three factors together explained 94.1% of the variation, with Bio6 having the greatest impact at 63.9%. Contribution rate analysis showed that temperature factors had the most significant influence on the distribution of the four *Taxus* species, followed by human activity and precipitation factors. Additionally, terrain, soil, and vegetation factors imposed varying constraints.

### 2.3. Spatial Distribution Patterns of Potentially Suitable Areas for Four Taxus Species Under the Current Climate Scenario

**Figure 1:**
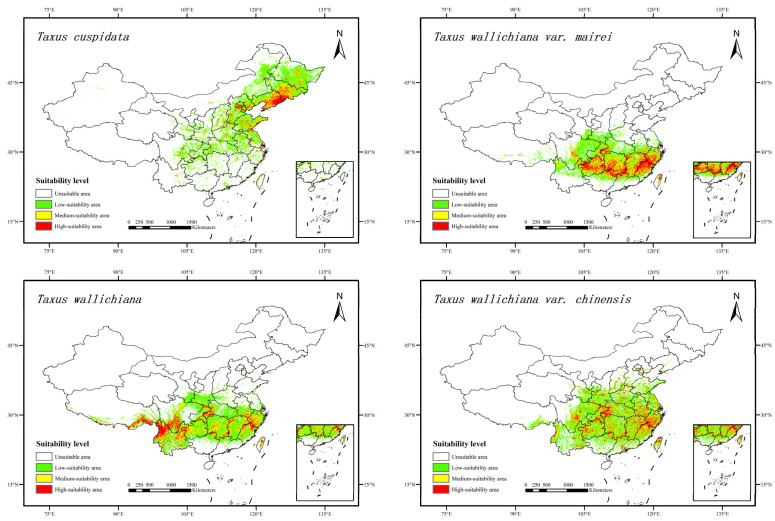
Potential habitat distribution of four *Taxus* species in China under current climate conditions.

According to MaxEnt model predictions, under the current climate scenario (Figure 1), *Taxus wallichiana* var. *chinensis* had the largest suitable habitat area among the four *Taxus* species, covering approximately 200.89 × 10^4^ km^2^, accounting for 21.17% of China’s land area. This was followed by *Taxus wallichiana*, with a suitable habitat area of about 179.18 × 10^4^ km^2^, representing 18.88% of China’s land area. The suitable habitat areas for *Taxus wallichiana* var. *mairei* and *Taxus cuspidata* were 146.62 × 10^4^ and 136.47 × 10^4^ km^2^, covering 15.45 and 14.38% of China’s land area, respectively (Table 3).

Under the current climate scenario, the highly suitable habitats for *Taxus cuspidata* were mainly found in eastern Liaoning Province and southern Jilin Province in China. Smaller areas were also present in Heilongjiang, Hebei, and Shandong, covering approximately 1.33% of the country’s land area. Moderately suitable habitats were primarily located in central Jilin, central Heilongjiang, and central Liaoning, comprising approximately 1.65% of the country’s land area. The distribution of *Taxus cuspidata*’s suitable habitat across China’s provincial regions is shown in Table 4. Heilongjiang had the largest suitable habitat area for *Taxus cuspidata*, measuring 20.490 × 10^4^ km^2^.

Under the current climate scenario, the highly suitable habitats for *Taxus wallichiana* var. *mairei* were mainly found in southern Hunan, western Guizhou, northern Fujian, Jiangxi, and southwestern Zhejiang, covering about 2.79% of the country’s land area. Moderately suitable habitats were primarily located in central Hunan, Jiangxi, and northern Guizhou, Sichuan, western Zhejiang, comprising approximately 3.35% of the country’s land area. The distribution of suitable habitats for *Taxus wallichiana* var. *mairei* across China’s provincial regions is shown in Table 5. Hunan had the largest suitable habitat area for *Taxus wallichiana* var. *mairei*, measuring 20.032 × 10^4^ km^2^.

Under the current climate scenario, the highly suitable habitats for *Taxus wallichiana* were mainly found in northwestern Yunnan, southeastern Tibet, and southern Sichuan, covering about 1.84% of the country’s land area. Moderately suitable habitats were mainly located in northeastern Yunnan, central Hunan, western Guizhou, Sichuan and Jiangxi, accounting for approximately 3.81% of the country’s land area. The distribution of suitable habitats for *Taxus wallichiana* across China’s provincial regions is shown in Table 6. Yunnan had the largest suitable habitat area for *Taxus wallichiana*, measuring 29.197 × 10^4^ km^2^.

Under the current climate scenario, the highly suitable habitats for *Taxus wallichiana* var. *chinensis* were mainly found in western Zhejiang, western Hubei, eastern Jiangxi, and central Sichuan, covering about 1.33% of the country’s land area. Moderately suitable habitats were mainly located in central Sichuan, Hunan, Jiangxi, eastern Yunnan, and Guizhou, covering approximately 3.26% of the country’s land area. The distribution of suitable habitats for *Taxus wallichiana* var. *chinensis* across China’s provincial regions is shown in Table 7. Sichuan had the largest suitable habitat area for *Taxus wallichiana* var. *chinensis*, measuring 21.863 × 10^4^ km^2^.

### 2.4. Characteristics of Potential Suitable Areas for Taxus Species Under Different Climate Scenarios

**Figure 2:**
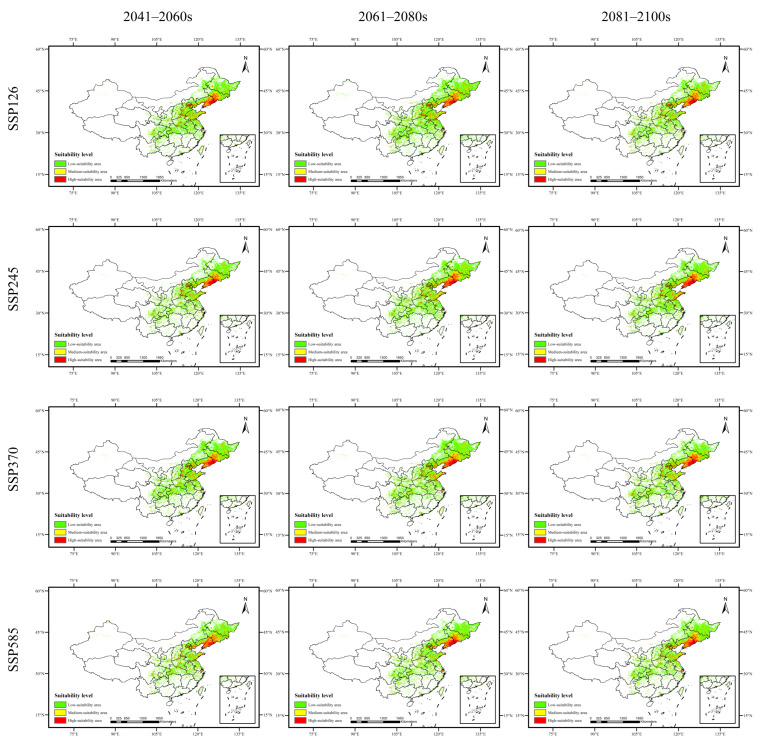
Potential distribution range of *Taxus cuspidata* in China under future climate scenarios.

**Figure 3:**
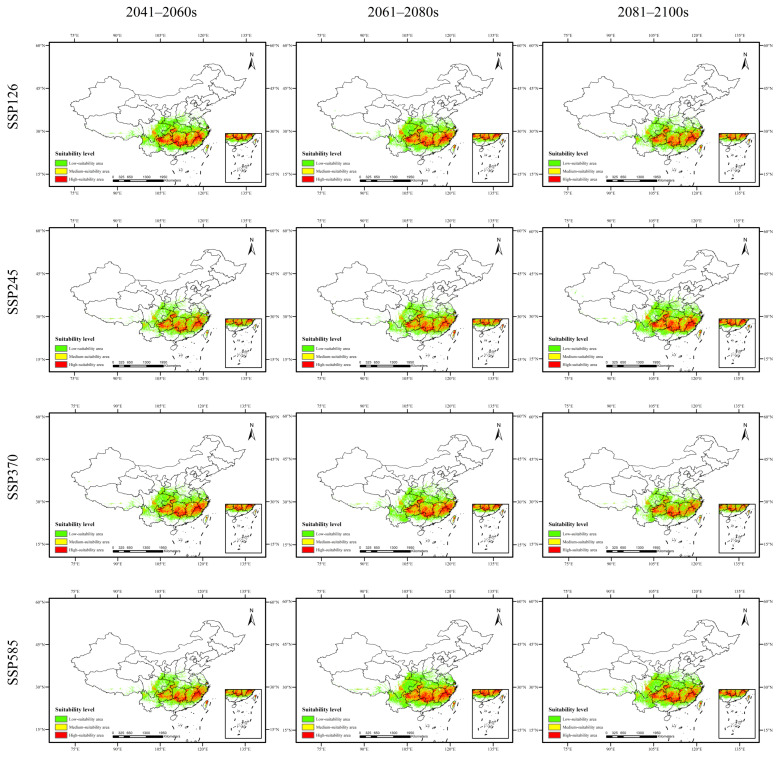
Potential distribution range of *Taxus wallichiana* var. *mairei* in China under future climate scenarios.

**Figure 4:**
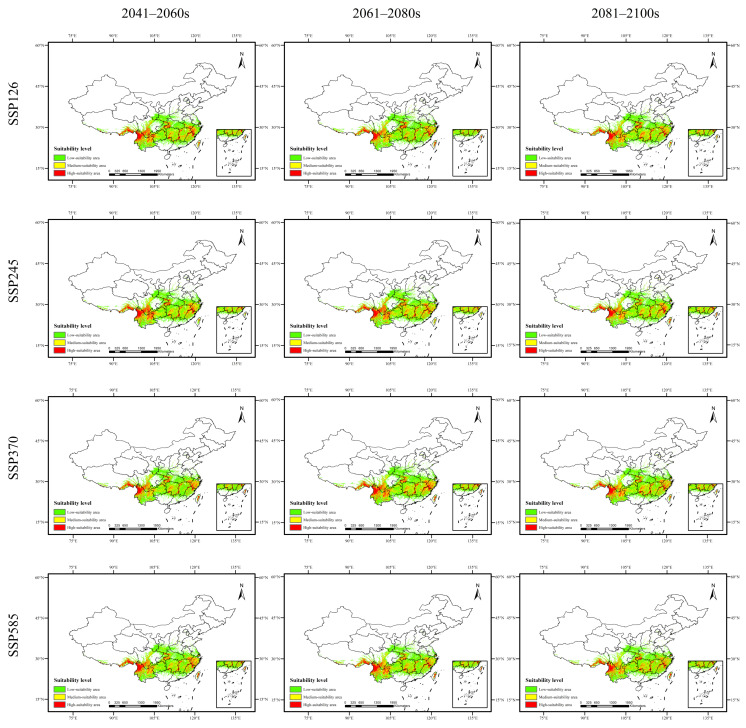
Potential distribution range of *Taxus wallichiana* in China under future climate scenarios.

**Figure 5:**
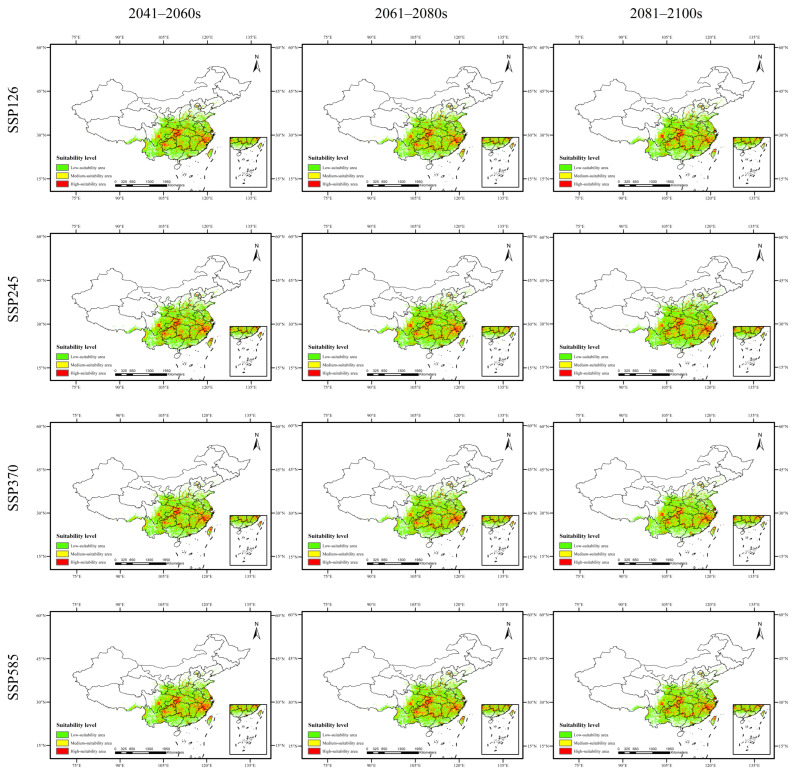
Potential distribution range of *Taxus wallichiana* var. *chinensis* in China under future climate scenarios.

As shown in Figure 2, Figure 3, Figure 4 and Figure 5 and Table 8, the changes in suitable habitat areas for *Taxus cuspidata* and *Taxus wallichiana* var. *mairei* under different future climate scenarios show marked differences, with both increases and decreases observed. Specifically, under the SSP585 climate scenario, the suitable habitat area for *Taxus cuspidata* exhibits a consistent shrinking trend across all three future periods (2050s, 2070s, 2090s). By the 2090s, its suitable area decreases under all three climate scenarios except SSP245. Under the SSP245 scenario, the most significant reduction occurs in the 2050s, with a total decrease of 20.141 × 10^4^ km^2^, primarily affecting western Heilongjiang Province, northwestern Jilin Province, and eastern Inner Mongolia. Conversely, under the SSP126 scenario, the most significant increase in suitable habitat area occurred in the 2070s, expanding by 23.357 × 10^4^ km^2^. The growth areas were mainly concentrated in eastern Heilongjiang Province, eastern Shandong Province, and southern Hebei Province. Under the SSP585 scenario, *Taxus wallichiana* var. *mairei* exhibits the largest increase in suitable habitat area during the 2070s, expanding by 39.254 × 10^4^ km^2^. The growth areas were primarily distributed in western Sichuan Province, southwestern Hubei Province, and northern Yunnan Province. Under the SSP370 scenario, the most pronounced reduction in suitable habitat area occurred in the 2090s, decreasing by 17.75 × 10^4^ km^2^. The areas experiencing reduction were primarily concentrated in eastern and central Sichuan Province. *Taxus wallichiana* and *Taxus wallichiana* var. *chinensis* generally exhibited increasing trends in suitable habitat area under most future climate scenarios. *Taxus wallichiana* exhibits area expansion across all scenarios in both the 2050s and 2090s, with sustained growth particularly evident in the SSP245 and SSP370 scenarios throughout future periods. The largest expansion occurs in the 2070s under SSP370, with an increase of 13.791 × 10^4^ km^2^ primarily distributed in northeastern Yunnan Province and western Hubei Province. Conversely, the most significant reduction in suitable habitat area under SSP585 occurs in the 2070s, with a decrease of 3.458 × 10^4^ km^2^, with the decline concentrated in eastern and southern Sichuan Province. For *Taxus wallichiana* var. *chinensis*, suitable habitat increased in all scenarios except SSP585, where it decreased by 1.917 × 10^4^ km^2^ in the 2090s. Under the SSP245 scenario, the suitable habitat area gradually increases over time, reaching its maximum expansion by the 2090s with an increase of 34.537 × 10^4^ km^2^. The primary areas of expansion are located in western and northern Sichuan Province, eastern Yunnan Province, and western Hunan Province.

### 2.5. Shift in the Centroid of Suitable Habitats for Four Taxus Species

**Figure 6:**
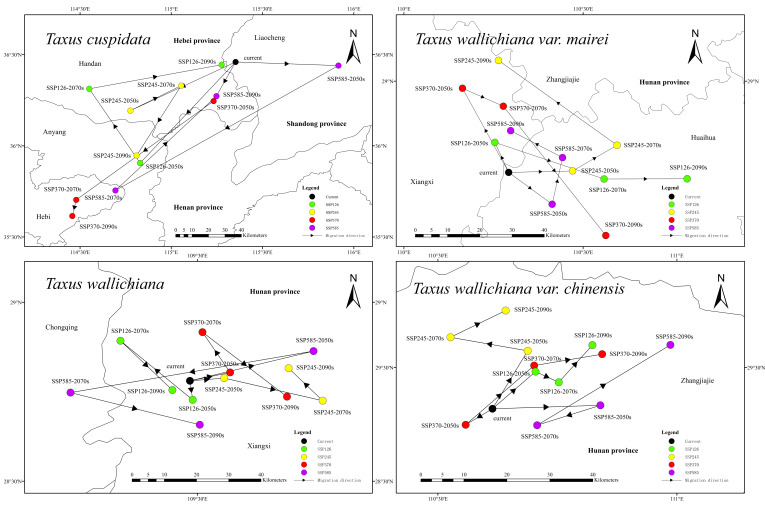
Changes in the centroid of suitable habitats for four species of the genus *Taxus* under climate change scenarios.

Figure 6 shows that under the current climate scenario, the center of gravity for the suitable habitat of *Taxus cuspidata* is located in the western region of Liaocheng City, Shandong Province. Across all future climate scenarios, it exhibits a trend of shifting southwestward. Specifically, under the SSP370 scenario, the center of gravity continuously shifts southwestward over time, reaching a migration distance of 124 km by the 2090s, and ultimately settling in the southeastern part of Hebi City, Henan Province, marking the period with the greatest migration distance and southernmost distribution in the future. Additionally, the future center of gravity for *Taxus cuspidata* is projected to occur in six climate scenarios within the northeastern region of Anyang City, Henan Province, while two climate scenarios project it being distributed in the southeastern part of Handan City, Hebei Province. Under current climate conditions, the suitable habitat center of *Taxus wallichiana* var. *mairei* is located in the northwest region of Huaihua City, Hunan Province. In most future climate scenarios, it exhibits a northward migration trend, which is most pronounced under the SSP245 scenario. The center gradually shifts northward over time, reaching its northernmost position in the northeast region of Xiangxi Tujia and Miao Autonomous Prefecture, Hunan Province, by the 2090s, with a migration distance of approximately 35 km. Under the SSP370 scenario, the center of suitability in the 2090s shifts southeastward by approximately 32.8 km. Furthermore, all other projected future centers of suitability for *Taxus wallichiana* var. *mairei* remain within the administrative boundaries of Huaihua City and Xiangxi Autonomous Prefecture in Hunan Province. Under current climate conditions, the suitable habitat center of *Taxus wallichiana* is located in the western region of Xiangxi Autonomous Prefecture, Hunan Province. Except for the SSP585 scenario, under which the center shifts westward by approximately 32.5 km to the eastern region of Chongqing Municipality by the 2070s, the center remains within Xiangxi Autonomous Prefecture under all other scenarios. Furthermore, the future center of suitability for *Taxus wallichiana* under SSP245 and SSP370 scenarios shows an overall eastward migration trend. Under current climate conditions, the center of suitable habitat for *Taxus wallichiana* var. *chinensis* is located in the central region of Zhangjiajie City, Hunan Province. In most future scenarios, it exhibits a northward migration trend; this trend is particularly pronounced under SSP126 and SSP245. Specifically, under the SSP245 scenario, the center of gravity continues to shift northward over time, reaching the northern part of Zhangjiajie City by the 2090s, with a migration distance of approximately 23.2 km. Under the remaining future scenarios, the center of gravity remains within the boundaries of Zhangjiajie City, Hunan Province.

## 3. Discussion

### 3.1. Reliability of MaxEnt Model Predictions Based on Parameter Optimization and Their Ecological Interpretability

The reliability of MaxEnt model predictions primarily depends on three key factors: model selection, sample size, and environmental variable selection [20]. The MaxEnt model has become the preferred method for simulating and predicting potential species habitat ranges because of its advantages, including maintaining high predictive accuracy even with small sample sizes [21] and strong stability [22]. It has shown excellent predictive performance in studies on the potential geographic distribution patterns of endangered species [23]. This study used the ENMeval package to optimize two MaxEnt model parameters, namely the regularization multiplier and feature combination, effectively reducing the risk of overfitting [24], improving prediction reliability, and resulting in smoother and more reasonable environmental response curves [25]. Regarding sample size, the collected distribution point data for *Taxus* species were adequate in quantity and representative of the spatial distribution, meeting the model’s data quality requirements [26]. This study constructs species distribution models based solely on distribution points within China, which may lead to underestimation of the species’ complete ecological niche, particularly for species with transboundary distributions. Given that the core objective of this research is to inform conservation strategies within China, the analysis relies exclusively on reliable wild distribution records from within China. Future studies incorporating global distribution data would facilitate the construction of more comprehensive ecological niche models, thereby reducing uncertainty in predictive outcomes. The selection of environmental variables thoroughly considered multidimensional habitat characteristics, including climate, soil, human activity intensity, vegetation type, and terrain. Climate and soil variables were screened by creating a correlation coefficient matrix between these factors. Variables with an absolute correlation coefficient ≥0.8 were retained based on their contribution to initial MaxEnt model calculations. This method effectively reduced redundant information among variables, enhanced the model’s ecological interpretability [22], and significantly improved the accuracy of MaxEnt predictions. The model’s prediction results showed AUC values exceeding 0.9, indicating high prediction accuracy [27]. Therefore, the optimized MaxEnt model can be used to forecast the changing distribution patterns of potential suitable habitats for the four *Taxus* species. This study employs the BCC-CSM2-MR single climate model for future distribution simulations. Although this model demonstrates satisfactory performance in simulating regional climate patterns across China, discrepancies persist in regional climate characteristics among different global climate models. Future adoption of multi-model ensemble methods would facilitate systematic assessment and mitigation of uncertainties inherent in single-model approaches, thereby enhancing the robustness of species distribution change projections.

### 3.2. Driving Factors of Geographic Distribution Patterns for Four Taxus Species Under Different Climate Scenarios

Based on the contribution rates, training gains, and single-factor response curves calculated by the model, the environmental factors influencing the geographic distribution patterns of four*Taxus* species were identified. Bio6 was the primary environmental factor affecting *Taxus* species, significantly impacting the potential geographic distribution of three species. As Tertiary relict plants, *Taxus* species are sensitive to extreme cold. The minimum temperature of the coldest month, a key climate variable reflecting winter severity and cold events, directly influenced the distribution of three *Taxus* species (excluding *Taxus cuspidata*). Its contribution to the potential ranges of *Taxus wallichiana* var. *mairei*, *Taxus wallichiana*, and *Taxus wallichiana* var. *chinensis* was 69.0, 66.7, and 63.9%, respectively. The single-factor contribution rate of the minimum temperature of the coldest month for these three species was higher than that of other environmental factors. Response curves for this factor showed ideal survival temperature ranges for the three plant species were −4.8 to 5.8, −6.4 to 5.4, and −7.8 to 8.0 °C, respectively. The importance of the minimum temperature of the coldest month as a limiting factor for the distribution of *Taxus* species has been supported by multiple studies [28,29]. For instance, the distribution of *Taxus wallichiana* in high-altitude areas is strictly limited by cold tolerance. Previous research [30] has shown that this species mainly occurs in Himalayan regions where winter temperatures stay above −8 °C, which aligns with the ideal survival temperature range found in this study’s response curve. Additionally, Chen et al. [31] analyzed the distribution of Chinese ferns and seed plants and concluded that the temperature in the coldest month was a key climate variable influencing rare plants, such as *Taxus wallichiana* var. *mairei*, in subtropical regions. Liu et al. [32] reviewed advances in Chinese plant phylogeography and found that the plant (Including *Taxus* species) distribution in China’s subtropical zones was generally limited by winter low temperatures. This supports the current study’s finding that the minimum temperature in the coldest month is a primary factor limiting the geographic distribution of *Taxus* species, confirming the reliability of this conclusion. This temperature dependence may be linked to the physiological traits of *Taxus* species. Low temperatures directly impact photosynthetic enzyme activity and cell membrane stability, and seedlings are particularly vulnerable to frost. Therefore, extreme winter cold is a key environmental constraint on their distribution.

Precipitation factors and human activities significantly influenced the geographic distribution of four *Taxus* species. Our results showed that Bio18 explained 17.4% of the variation in the distribution of *Taxus cuspidata*. This supports Chen et al.’s conclusion that precipitation in the warmest month was the main climatic factor affecting *Taxus cuspidata*’s potential range. The annual mean precipitation (Bio12) contributed 5.8% to the variation in the distributions of *Taxus wallichiana* var. *chinensis*. Precipitation in the driest month (Bio14) accounted for 7% of the variation in *Taxus wallichiana* var. *mairei*’s distribution. In China, due to the medicinal value of *Taxus* species, excessive logging and paclitaxel extraction have severely harmed natural populations through human activities. This has caused an ongoing decline in population stability and a decrease in suitable habitats. The IPCC Sixth Assessment Report states that human activities are now the main threat to rare and endangered plants. Land-use changes, overexploitation, and climate change have caused the loss of 47% of global plant habitats, accelerating species extinction by 100–1000 times compared to natural rates. For example, *Taxus cuspidata*, a relict plant that has survived until today, faces intense human disruption, threatening its population stability [33]. The cause of this phenomenon may lie in the fact that the primary suitable habitat for *Taxus cuspidata* is currently concentrated in Northeast China, a region that serves as a vital agricultural, forestry, and industrial base for the country. Long-term, large-scale land reclamation, forest harvesting, and urbanization have directly led to the loss and severe fragmentation of its native habitat. Furthermore, as a shade-tolerant species, *Taxus cuspidata* exhibits slow growth and weak natural regeneration capacity [13], further diminishing its resilience to habitat disturbance. Human activities not only directly remove individual plants but also disrupt local microclimates and block seed dispersal pathways, making it difficult for the species to maintain stable populations and expand within fragmented landscapes. Human disturbance varied among the four *Taxus* species, with *Taxus cuspidata* most affected by the intensity of human activity, contributing 39.1%. This was followed by *Taxus wallichiana* var. *chinensis* at 24.4%. Conversely, *Taxus wallichiana*, and *Taxus wallichiana* var. *mairei* showed less sensitivity to human activity, each with contribution rates below 5%, indicating that they may tolerate certain levels of human disturbance.

Additionally, soil, topography, and vegetation factors also significantly influenced the geographic distribution patterns of the four *Taxus* species. Among these, slope accounted for 7.1% of the variation in the distribution of *Taxus wallichiana*, and elevation explained 4.8% of that for *Taxus cuspidate*. Of the soil factors, only the contribution rate of T_BS was influential, affecting *Taxus cuspidata*, and *Taxus wallichiana* var. *mairei* with contribution rates of 5.5 and 4.8%. The contribution of vegetation type factors to the geographic distribution of all four species was less than 2%. In conducting future distribution projections, this study assumes that non-climatic environmental factors such as soil properties and vegetation types will remain constant due to limitations in currently available data. However, against the backdrop of long-term climate change, land use patterns, vegetation community structures, and soil development processes may undergo dynamic evolution. For instance, future warming may trigger altitudinal shifts in mountain vegetation, thereby further influencing local soil organic matter content and moisture conditions. Neglecting these dynamic interactions may reduce the reliability of long-term predictions for species’ suitable habitats. Furthermore, the model does not yet incorporate biotic and abiotic factors such as interspecific competition, pest and disease spread, and small-scale microhabitat heterogeneity, which may also significantly influence actual species distribution patterns. Future research could explore developing predictive models that integrate dynamic changes in both soil and vegetation to enhance the ecological realism of distribution simulations.

### 3.3. Changes in the Geographic Distribution Patterns of Four Taxus Species Under Different Future Climate Scenarios

Climate significantly influences plant growth and reproduction, acting as the main factor determining the geographic distribution of different plant species [34]. This study on predicting habitat suitability for four *Taxus* species showed that each species had its own distinct geographic distribution pattern because of differences in habitat suitability. The suitable habitat for *Taxus cuspidata* was mainly in northeast China, with additional ranges in north China and east China. This finding agrees largely with the potential habitat predictions for *Taxus cuspidata* made by the MaxEnt model, as reported by Chen et al. [17]. In the analysis by Chen et al., low, medium, and high suitability zones for *Taxus cuspidata* covered 17.6 × 10^5^, 7.7 × 10^5^, and 3.3 × 10^5^ km^2^, respectively, totaling 28.6 × 10^5^ km^2^ of potential suitable habitat. This differed from the 13.647 × 10^5^ km^2^ of suitable area found in this study. The difference may be due to variations in the environmental variables. Building upon Chen et al.’s primary climate-variable-based predictions, this study further integrates multidimensional environmental factors —including soil, topography, human footprint, and vegetation types—to construct an ecological constraint model that more closely approximates real habitats. Consequently, the resulting suitable habitat area is smaller. The potential distribution patterns of *Taxus cuspidata* remain relatively stable under various future climate scenarios; however, the suitable area fluctuates across different time periods and scenarios, indicating that this species has relatively limited adaptability to climate change. The potential habitat for *Taxus wallichiana* var. *mairei* was mostly located in southern regions, covering east, central, and southwest China, aligning with findings by Li Yanhong et al. [35], who used the MaxEnt model. Their results showed low fitness areas of *Taxus wallichiana* var. *mairei* in China’s subtropical warm-temperate monsoon zone and high fitness areas in the Qinling-Daba Mountains, consistent with this study. The potential distribution of *Taxus wallichiana* var. *mairei* under different future climate scenarios shows overall consistency. The center of gravity of its distribution exhibits a northward migration trend under most climate scenarios, though the migration range remains confined to Huaihua City and Xiangxi Tujia and Miao Autonomous Prefecture in Hunan Province. Under the SSP245 scenario, the center of gravity shifts northward overall, whereas under SSP370, it first moves northward and then southward. However, under SSP585, the migration trend reverses. Under most scenarios, the total suitable habitat area for *Taxus wallichiana* var. *mairei* increases, indicating its potential adaptability to future climate conditions. This may stem from the species’ preference for warm, humid environments: the temperature rise caused by future warming remains within its tolerance range, limiting the negative impacts of climate warming and offering opportunities for expansion into new suitable regions. The potential suitable habitats for *Taxus wallichiana* are primarily distributed in East China, the Tibet Autonomous Region in Southwest China, and surrounding areas. This aligns with the findings of Shu et al. [19], who reported that *Taxus wallichiana* was mainly distributed in the southeastern region of Tibet, extending eastward from the northern foothills of the Himalayas to the Hengduan Mountains. Under different future climate scenarios, the distribution of *Taxus wallichiana*’s potential suitable habitat remains relatively stable, with its centroid consistently fluctuating within the Xiangxi Autonomous Prefecture of Hunan Province and showing no significant north–south migration. Under SSP245 and SSP370 scenarios, the centroid generally shifted eastward, while the opposite trend was observed under SSP126. Furthermore, the total suitable area showed an increasing trend under most climate scenarios, indicating that the potential suitable habitat of *Taxus wallichiana* could also exhibit strong adaptability to future climate conditions.

Under different future climate conditions, the centers for *Taxus wallichiana* var. *chinensis* generally shifted toward higher latitudes over time. Considering global warming, existing research has shown that potential suitable habitats of many species are expanding toward higher latitudes, reflecting plants’ adaptation to climate change through an altered geographic distribution [36]. *Taxus cuspidata* faces a significant risk of habitat degradation under various future climate scenarios, especially under the SSP245 scenario in the 2070s, when it experiences the greatest loss of suitable habitat and severe habitat fragmentation. This indicates that both climate change and human activities pose significant threats to the future suitable range of *Taxus cuspidata*. Based on research on the relationship between *Taxus cuspidata*’s radial growth and climate and its population endangerment mechanisms, Zhou et al. [37] suggested that the species’ endangered status likely results from the combined effects of human and climatic factors, with human factors, including habitat destruction and overharvesting, playing a more critical role. Therefore, measures such as artificial tending, establishing protected sub-areas, and strengthening management will be more effective for its conservation [38]. This study predicted suitable habitats for four *Taxus* species under current and future climate scenarios using 40 environmental factors. However, other factors such as deep soil properties, ultraviolet radiation, and interspecific interactions may also influence the distribution patterns of *Taxus* species. Future research should include additional environmental variables such as deep soil factors and ultraviolet radiation to offer more robust scientific foundations for *Taxus* conservation efforts.

## 4. Materials and Methods

### 4.1. Data Sources and Processing

**Figure 7:**
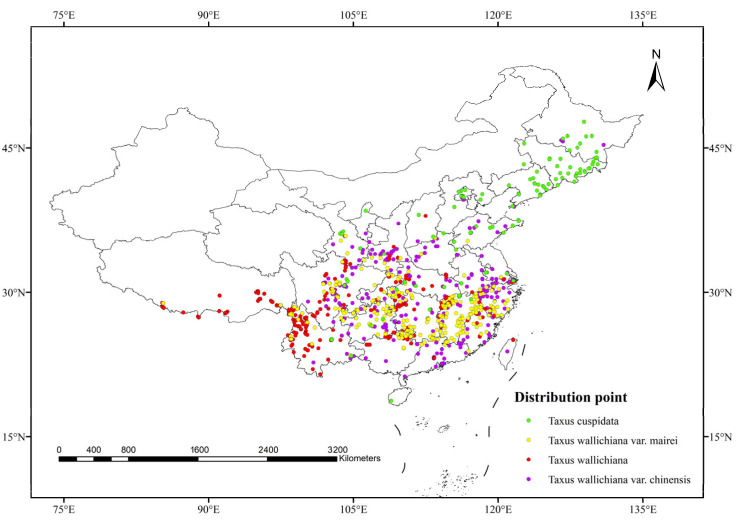
Distribution of valid occurrence records for four *Taxus* species used in MaxEnt modeling.

The sources of the species distribution point data used in this study are listed in Table 9. Geographic coordinates for the four *Taxus* species were obtained from the Global Biodiversity Information Facility (https://www.gbif.org/ accessed on 20 January 2025), the China Virtual Herbarium (https://www.cvh.ac.cn/ accessed on 23 January 2025), the National Specimen Information Infrastructure (http://www.nsii.org.cn/ accessed on 25 January 2025), iPlant (https://www.iplant.cn/ accessed on 27 January 2025), and relevant literature. A total of 2410 distribution point records for the four *Taxus* species within China were collected and compiled. Data with recorded latitude and longitude were used directly. For distribution points with only location information, the Baidu Coordinate Retrieval System (https://api.map.baidu.com/lbsapi/getpoint/ accessed on 10 March 2025) was used to extract the latitude and longitude data, and excluded records explicitly identified as related to artificial cultivation. According to the software requirements for constructing species distribution models, first exclude missing, invalid, and duplicate latitude and longitude records. Then import the environmental variables and filtered distribution points into R 4.3.3 to remove redundant distribution data, and a 1 km × 1 km grid was created to ensure only one distribution point per grid cell, preventing model overfitting. This resulted in 1179 valid distribution points (Figure 7): 117 for *Taxus cuspidata*, 294 for *Taxus wallichiana* var. *mairei*, 391 for *Taxus wallichiana*, and 377 for *Taxus wallichiana* var. *chinensis*. Following the input requirements specified in MaxEnt 3.4.4 documentation, the data were organized into CSV format for subsequent analysis.

The environmental variables used in this study included climate, soil, topography, Hfp, and vegetation type data. Climate and topography data were obtained from the Global Climate Database (https://www.worldclim.org/) at a spatial resolution of 30 arc- seconds. Nineteen bioclimatic variables and elevation data were downloaded from the WorldClim database (Table 10). Topography data were derived using spatial analysis tools in ArcGIS 10.8, specifically the surface analysis tool to extract slope and aspect from elevation data. Current climate data were from WorldClim version 2.1 (1970–2000). Future climate data were obtained from the Beijing Climate Center Medium Resolution Climate System Model (BCC-CSM 2-MR), part of the Sixth Coupled Model Intercomparison Project, which is most suitable for Chinese climate change research [39]. Future climate periods included the 2050s (2041–2060), 2070s (2061–2080), and 2090s (2081–2100), with bioclimatic variables segmented accordingly. Four shared socioeconomic pathways (SSPs) defined the climate change scenarios: SSP126, representing low vulnerability, low mitigation pressure, and low radiative forcing; SSP245, combining moderate societal vulnerability with moderate radiative forcing; SSP370, with high societal vulnerability and relatively high anthropogenic radiative forcing; and SSP585, representing a high forcing scenario [40]. Soil data were sourced from the Harmonized World Soil Database (https://www.fao.org/soils-portal/data-hub/soil-maps-and-databases/harmonized-world-soil-database-v12/en/ accessed on 20 January 2025) with a spatial resolution of 30 arc-seconds, utilizing 16 topsoil data points (Table 11). Hfp data were obtained from https://www.earthdata.nasa.gov/data/catalog/sedac-ciesin-sedac-lwp2-hf-geog-2.0/, with a spatial resolution of 1 km. Vegetation type data were obtained from the Resource and Environment Science and Data Center (https://www.resdc.cn/) with a 1 km spatial resolution. All environmental data were processed using ArcGIS 10.8, unified into the WGS–1984 coordinate system, and resampled to a consistent spatial resolution of 30 arc-seconds. Based on China’s administrative boundary map data (GS(2024) 0650), each dataset was clipped according to administrative boundaries and saved in ASC format for use in MaxEnt 3.4.4.

### 4.2. Environment Variable Filtering

**Figure 8:**
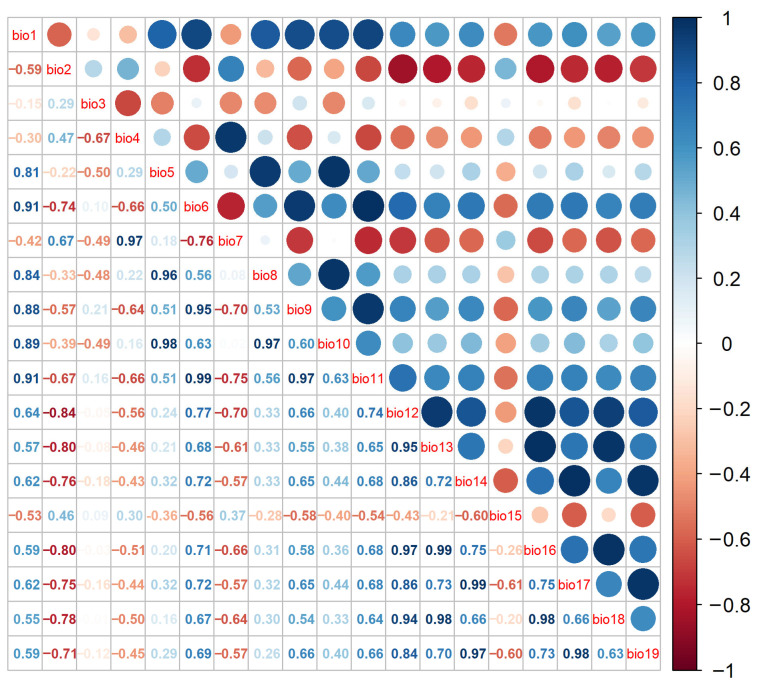
Correlation analysis of 19 climatic variables.

**Figure 9:**
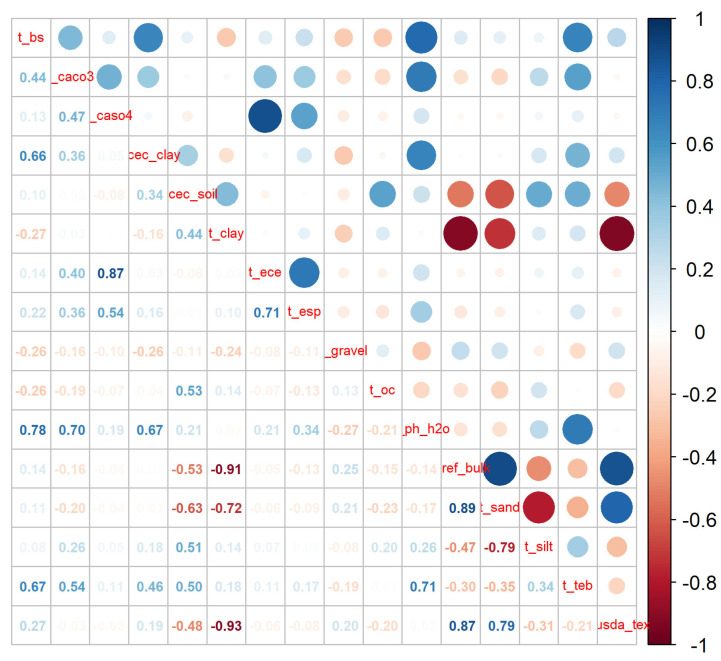
Correlation analysis of 16 soil variables.

Due to the high correlation among environmental variables, using all variables in model predictions could lead to overfitting. Therefore, the selected environmental variables were screened. Using the MaxEnt 3.4.4 model, 10 iterations were conducted on the chosen 40 environmental variables to develop an initial model. Correlation analysis was performed with R 4.3.3 on 19 climate variables and 16 soil variables (Figure 8 and Figure 9). As can be seen from the figure, larger and darker blue circles indicated a stronger positive correlation between environmental factors, while larger and darker red circles indicated a stronger negative correlation. For pairs of variables with absolute correlation coefficients ≥0.8, we retained the variable with the higher contribution rate in the initial MaxEnt model (using all variables) and excluded the other variable. After this step, we reran the model using the remaining, less correlated set of variables. Finally, from this set, we further excluded variables with a contribution rate below 1% in the final model (Table 12).

### 4.3. MaxEnt Model Construction and Parameter Optimization

This study used the ENMeval package in R 4.3.3 to identify the best parameter combinations. By optimizing the regularization multiplier and feature classes, it balanced model complexity and reduced overfitting, thus improving prediction accuracy. The MaxEnt model features included linear (L), quadratic (Q), hinge (H), product (P), and threshold (T). The package set regularization values of 0.5–4 in steps of 0.5, resulting in eight coefficients. Six feature combinations—L, H, LQ, LQH, LQHP, and LQHPT—were tested. The incremental ΔAICc correction method evaluates model complexity and fit, with the model having the smallest ΔAICc usually considered optimal [41]. When running the MaxEnt model, use the optimized regularization multiplier and feature combination parameters. Allocate 25% of known distribution points as a random test dataset, with the output format set to Cloglog. Run the model 10 times iteratively using cross-validation as the iteration method, with a maximum background point count of 10,000. Additionally, the Jackknife and response curve functions were enabled, with all other parameters left at their default settings. Model performance was evaluated by employing the area under the receiver operating characteristic curve (AUC) and true skill statistic (TSS). The AUC ranges from 0 to 1, with values closer to 1 indicating higher accuracy. Typically, an AUC of 0.6–0.7 indicates poor performance, 0.7–0.8 indicates moderate performance, 0.8–0.9 indicates good performance, and 0.9–1.0 indicates excellent performance [42]. The values of TSS range from −1 to 1, where positive values approaching 1 indicate a high relationship between the predictive model and the distribution, and negative values reflect a poor relationship [43].

### 4.4. Habitat Suitable Area Delineation and Centroid Migration Analysis

Using the “Reclassify” tool in ArcGIS 10.8, suitability index *p* values ranging from 0 to 1 were categorized. Liu et al. [44] demonstrated that the maximum sensitivity plus specificity (MaxSSS) was the most robust threshold selection method with the best overall performance. This study employs MaxSSS as the threshold to classify model-predicted potentially suitable areas into suitable zones (*p* ≥ MaxSSS) and non-suitable zones (*p* < MaxSSS). To further differentiate suitability levels, the natural breaks method is applied to subdivide suitable zones into three tiers: low suitability, medium suitability, and high suitability. To quantify the trends in suitable habitat area and spatial patterns for the four *Taxus* species under different climate scenarios, ArcGIS 10.8 software was used to calculate both the suitable habitat area and its proportion relative to the total study area for each climate scenario, thereby assessing the magnitude of area changes. Furthermore, to reveal the spatial migration characteristics of suitable areas, the continuous suitable area raster data output from the MaxEnt model was uniformly converted into a binary file (suitable area = 1, unsuitable area = 0) using the MaxSSS threshold. Subsequently, the “Mean Center” tool was used to calculate the centroid coordinates of suitable areas for each period. By connecting the center-of-mass points across different periods, spatial migration paths were constructed. Based on this, migration distance and direction were calculated.

## 5. Conclusions

This study employed an optimized MaxEnt model based on the ENMeval package to simulate and predict the potentially suitable habitats of four *Taxus* species under current and future climate scenarios. The optimized MaxEnt model achieved high predictive accuracy, with AUC values exceeding 0.9 and TSS values surpassing 0.7, enabling reliable assessment of habitat suitability and distribution shifts. Temperature, particularly the minimum temperature of the coldest month, was identified as the dominant environmental factor influencing the distribution of most *Taxus* species, with the exception of *Taxus cuspidata*, whose distribution was most significantly affected by human activity. Under most future climate scenarios, *Taxus wallichiana* and *Taxus wallichiana* var. *chinensis* exhibited an overall expansion trend in suitable habitats and a northward shift in their distribution centers, indicating strong adaptive potential, though their ranges remained within Hunan Province. In contrast, *Taxus cuspidata* and *Taxus wallichiana* var. *mairei* showed greater fluctuations in suitable habitat area, characterized by a mixed trend of expansion and contraction, along with a southward shift in their distribution centers, reflecting their vulnerability to future climate change and anthropogenic pressures. These findings provide a crucial scientific basis for the conservation and sustainable utilization of *Taxus* species in China, including the identification of priority areas for in situ protection, guidance for ex situ conservation and artificial cultivation site selection, and support for adaptive management strategies in response to climate change. Future research should further integrate dynamic ecological processes—such as interspecific competition and soil-vegetation feedbacks—to enhance the ecological realism of species distribution models.

## Figures and Tables

**Table 1:** ENMeval optimization results for MaxEnt model parameters.

Species Name	RM	FC	ΔAICc	Mean OR10	AUC	TSS
*Taxus cuspidata*	4	LQHPT	0.000	0.281	0.931	0.784
*Taxus wallichiana* var. *mairei*	0.5	LQ	0.000	0.128	0.930	0.819
*Taxus wallichiana*	0.5	LQ	0.000	0.109	0.929	0.775
*Taxus wallichiana* var. *chinensis*	0.5	LQ	0.000	0.140	0.903	0.725

Notes: RM, regularization multiplier; FC, feature class; Mean OR10: 10% omission rate; AUC, area under the curve; TSS, true skill statistic.

**Table 2:** Contribution rates of environmental factors influencing the distribution of four *Taxus* species.

Species Name	Environmental Variable	Percent Contribution (%)
*Taxus cuspidata*	Hfp, Bio18, Bio4, T_BS, Bio2, Elevation, Bio3, Bio9, Bio15, Slope, Vegetation, T_CaCO_3_, T_pH_H_2_O	39.1, 17.4, 11.6, 5.5, 5.2, 4.8, 4.7, 2.6, 2.3, 1.4, 1.4, 1.1, 1
*Taxus wallichiana* var. *mairei*	Bio6, Bio14, Bio8, T_BS, Bio2, Hfp, Bio3, Vegetation, Elevation, Aspect	69, 7.0, 4.9, 4.8, 3.7, 3.3, 2.2, 1.9, 1.4, 1.1
*Taxus wallichiana*	Bio6, Bio8, Slope, Hfp, Bio7, Vegetation, Bio12, Bio15, Elevation, Bio19, Aspect	66.7, 11.3, 7.1, 4.5, 2.8, 1.7, 1.3, 1.2, 1.2, 1, 1
*Taxus wallichiana* var. *chinensis*	Bio6, Hfp, Bio12, Slope, Aspect, Elevation, Bio4	63.9, 24.4, 5.8, 1.9, 1.4%, 1.4, 1.2

**Table 3:** Habitat suitability area of four *Taxus* species under current climate conditions (10^4^ km^2^).

Species Name	Low Suitability Area	Medium Suitability Area	High Suitability Area	Total Suitable Area
*Taxus cuspidata*	81.53	36.90	18.03	136.47
*Taxus wallichiana* var. *mairei*	61.97	49.94	34.70	146.62
*Taxus wallichiana*	87.99	58.51	32.67	179.18
*Taxus wallichiana* var. *chinensis*	100.12	66.99	33.78	200.89

**Table 4:** Province-level estimate of the suitable habitat area for *Taxus cuspidata* (10^4^ km^2^).

Province Name	Low Suitability Area	Medium Suitability Area	High Suitability Area	Total Suitable Area
Heilongjiang	14.821	4.651	1.017	20.490
Jilin	5.735	6.511	3.678	15.926
Liaoning	2.834	4.576	4.927	12.338
Shandong	5.559	4.319	1.636	11.515
Hebei	6.097	2.943	1.426	10.467
Inner Mongolia	7.527	1.642	0.246	9.416
Henan	4.922	1.497	0.554	6.974
Sichuan	4.869	1.370	0.406	6.646
Hubei	4.429	1.219	0.327	5.976
Anhui	3.980	1.183	0.388	5.552

**Table 5:** Province-level estimate of the suitable habitat area for *Taxus wallichiana* var. *mairei* (10^4^ km^2^).

Province	Low Suitability Area	Medium Suitability Area	High Suitability Area	Total Suitable Area
Hunan	4.547	8.796	6.688	20.032
Sichuan	12.110	4.886	1.333	18.330
Guizhou	2.798	7.211	6.489	16.500
Jiangxi	3.905	7.273	4.798	15.977
Fujian	1.829	3.066	4.930	9.825
Hubei	5.718	2.608	1.303	9.630
Yunnan	6.031	2.638	0.906	9.576
Zhejiang	2.093	3.620	3.564	9.277
Guangxi	3.584	2.313	1.767	7.664
Chongqing	3.226	2.492	0.819	6.537

**Table 6:** Province-level estimate of the suitable habitat area for *Taxus wallichiana* (10^4^ km^2^).

Province Name	Low Suitability Area	Medium Suitability Area	High Suitability Area	Total Suitable Area
Yunnan	9.628	10.294	9.275	29.197
Hunan	10.334	7.502	2.237	20.073
Sichuan	8.387	6.112	4.102	18.602
Jiangxi	7.935	6.045	1.916	15.898
Guizhou	7.865	6.281	1.428	15.575
Tibet	4.711	3.204	4.166	12.082
Hubei	7.146	2.855	2.000	12.002
Zhejiang	3.014	4.035	2.315	9.364
Fujian	3.697	3.494	1.813	9.005
Shaanxi	5.940	1.461	0.246	7.648

**Table 7:** Province-level estimate of the suitable habitat area for *Taxus wallichiana* var. *chinensis* (10^4^ km^2^).

Province Name	Low Suitability Area	Medium Suitability Area	High Suitability Area	Total Suitable Area
Sichuan	11.604	7.559	2.698	21.863
Yunnan	10.520	5.755	1.823	18.099
Hunan	8.470	6.060	2.660	17.190
Jiangxi	6.073	6.019	3.224	15.317
Hubei	6.543	5.408	3.258	15.210
Guizhou	7.046	5.578	2.519	15.144
Anhui	4.839	3.521	2.025	10.386
Henan	5.928	2.579	1.070	9.578
Fujian	3.104	3.825	2.326	9.256
Zhejiang	2.372	3.342	3.463	9.178

**Table 8:** Total habitat suitability area of four *Taxus* species under future climate conditions (10^4^ km^2^).

SSPs	Period	*Taxus cuspidata*	*Taxus wallichiana* var. *mairei*	*Taxus wallichiana*	*Taxus wallichiana* var. *chinensis*
SSP126	50s	137.85	170.20	183.70	215.81
	70s	159.82	161.46	178.74	212.41
	90s	124.48	142.17	179.51	214.18
SSP245	50s	116.32	138.98	186.00	223.35
	70s	141.85	144.29	183.26	230.20
	90s	141.50	170.85	180.51	235.43
SSP370	50s	144.21	153.19	180.01	212.52
	70s	150.09	159.09	192.97	210.26
	90s	130.52	128.87	186.88	208.31
SSP585	50s	118.43	139.77	184.30	215.33
	70s	122.22	185.88	175.72	215.36
	90s	129.08	178.12	180.67	198.97

**Table 9:** Data sources.

Data Source	Data Type	Website
Global Biodiversity Information Facility	Species distribution points	https://www.gbif.org/
Chinese Virtual Herbarium	Species distribution points	https://www.cvh.ac.cn/
National Specimen Information Infrastructure	Species distribution points	http://www.nsii.org.cn/
iPlant	Species distribution points	https://www.iplant.cn/
WorldClim	Climate data	https://www.worldclim.org/
	Terrain data	https://www.worldclim.org/
HWSD1.2	Soil data	https://www.fao.org/
NASA-Earthdata	Human footprint data	https://www.earthdata.nasa.gov/
Resource and Environmental Science Data Platform	Vegetation type data	https://www.resdc.cn/
National Platform for Common GeoSpatial Information Services	Map	https://www.tianditu.gov.cn/

**Table 10:** Climate environmental variables.

Environment Variables	Variable Description	Unit
Bio1	Annual mean temperature	°C
Bio2	Mean diurnal range	°C
Bio3	Isothermality	-
Bio4	Temperature seasonality	-
Bio5	Max temperature in the warmest month	°C
Bio6	Min temperature in the coldest month	°C
Bio7	Annual temperature range	°C
Bio8	Mean temperature of the wettest quarter	°C
Bio9	Mean temperature of the driest quarter	°C
Bio10	Mean temperature of the warmest quarter	°C
Bio11	Mean temperature of the coldest quarter	°C
Bio12	Annual precipitation	mm
Bio13	Precipitation in the wettest month	mm
Bio14	Precipitation in the driest month	mm
Bio15	Precipitation seasonality	-
Bio16	Precipitation in the wettest quarter	mm
Bio17	Precipitation in the driest quarter	mm
Bio18	Precipitation in the warmest quarter	mm
Bio19	Precipitation in the coldest quarter	mm
Elev	Elevation	m
Aspect	Aspect	°
Slope	Slope	%

**Table 11:** Soil environmental variables.

Environment Variables	Variable Description	Unit
T-gravel	Topsoil gravel content	% vol
T-silt	Topsoil silt fraction	% weight
T-clay	Topsoil clay fraction	% weight
T-REF-bulk	Topsoil reference bulk density	kg/dm^3^
T-OC	Topsoil organic carbon	% weight
T-pH-H_2_O	Topsoil pH (H_2_O)	−log(H+)
T-CEC-soil	Topsoil cation exchange capacity of soil	cmol/kg
T-CaSO_4_	Topsoil gypsum	% weight
T-ESP	Topsoil sodicity (ESP)	%
T_SAND	Topsoil sand fraction	% weight
T_USDA_TEX_CLASS	Topsoil USDA texture classification	-
T_CEC_CLAY	Topsoil CEC (clay)	cmol/kg
T_CaCO_3_	Topsoil calcium carbonate	% weight
T_BS	Topsoil base saturation	%
T_ECE	Topsoil salinity (Elco)	dS/m
T_TEB	Topsoil TEB	cmol/kg

**Table 12:** Environmental variable screening.

Environment Variable	Description	Unit	*Taxus cuspidata*	*Taxus wallichiana* var. *mairei*	*Taxus wallichiana*	*Taxus wallichiana* var. *chinensis*
Bio2	Mean diurnal range	°C	√	√		
Bio3	Isothermality	-	√	√	√	
Bio4	Temperature seasonality	-	√			√
Bio6	Min temperature of the coldest month	°C		√	√	√
Bio7	Annual temperature range	°C		√	√	
Bio8	Mean temperature of the wettest quarter	°C		√	√	
Bio9	Mean temperature of the driest quarter	°C	√			
Bio12	Annual precipitation	mm			√	√
Bio14	Precipitation in the driest month	mm		√		
Bio15	Precipitation seasonality	-	√		√	
Bio18	Precipitation in the warmest quarter	mm	√			
Bio19	Precipitation in the coldest quarter	mm			√	
T_BS	Topsoil base saturation	%	√	√	√	√
T-ESP	Topsoil sodicity (ESP)	%	√			
T_CaCO_3_	Topsoil calcium carbonate	% weight	√			
T-pH-H_2_O	Topsoil pH (H_2_O)	−log(H+)	√			
T_TEB	Topsoil TEB	cmol/kg	√			
T_USDA_TEX_CLASS	Topsoil USDA texture classification	-	√			
Elevation	Elevation	m	√	√	√	√
Aspect	Aspect	°	√	√	√	√
Slope	Slope	%	√	√	√	√
Hfp	Human footprint	-	√	√	√	√
Vegetation	Vegetation type	-	√	√	√	

Notes: The "√" indicates the environmental variables used for modeling.

## Data Availability

The data used in this study are available from the following sources. Global Biodiversity Information Facility: https://www.gbif.org/ (accessed on 20 January 2025), China Virtual Herbarium: https://www.cvh.ac.cn/ (accessed on 23 January 2025), National Specimen Information Infrastructure: http://www.nsii.org.cn/ (accessed on 25 January 2025), Plant Wisdom: https://www.iplant.cn/ (accessed on 27 January 2025), Global Climate Database: https://www.worldclim.org/ (accessed on 20 January 2025), Harmonized World Soil Database: https://www.fao.org/ (accessed on 21 January 2025), Human footprint data: https://www.earthdata.nasa.gov/data/catalog/sedac-ciesin-sedac-lwp2-hf-geog-2.0/ (accessed on 5 February 2025), Resource and Environment Science and Data Center: https://www.resdc.cn/ (accessed on 10 February 2025), DOI: 10.6084/m9.figshare.30509525.

## Abbreviations

The following abbreviations are used in this manuscript:

FC	Feature Classes
RM	Regularization Multiplier
AUC	Area Under the Curve
ROC	Receiver Operating Characteristic Curve
ΔAICc	Difference in Akaike Information Criterion corrected
OR10	10% Omission Rate
